# Intraarticular gold for knee osteoarthritis: An ancillary analysis of biomarkers and outcome of a pilot study

**DOI:** 10.1016/j.ocarto.2024.100514

**Published:** 2024-08-31

**Authors:** Sten Rasmussen, Christopher Aboo, Jacob Skallerup, Allan Stensballe

**Affiliations:** a Department of Orthopedic Surgery, Sport and Arthroscopy, Aalborg University Hospital, Denmark; b Department of Clinical Medicine, Aalborg University, Denmark; c Department of Health Science and Technology, Aalborg University, Aalborg, Denmark; d Clinical Cancer Research Center, Aalborg University Hospital, Aalborg, Denmark; e Sino-Danish Center for Education and Research, University of Chinese Academy of Sciences, Beijing, China

**Keywords:** Osteoarthritis, Omics, Gold, Inflammation, Pain, Outcome prediction

## Abstract

**Objective:**

In a previous pilot study, we have shown that intraarticular gold micro-particles can reduce knee osteoarthritis (KOA) pain at two years follow-up. Proteomic changes in serum and synovial fluid within eight weeks were associated with multiple inflammatory and immunological processes. The relation between the different biomarkers and the outcome measures is not known. We hypothesized that improvement in pain and function were associated with specific groups of biomarkers. We present the integrative analyses between proteomic biomarkers and outcomes.

**Design:**

A cohort of 30 patients, with moderate KOA, were included. Using the patients’ synovial fluid as the carrier, 20 ​mg gold microparticles were injected intraarticularly. Clinical outcome measures at inclusion, 8 weeks, and 2 years, were the PainDetect questionnaire, WOMAC pain, stiffness, and function. In addition, Quantitative Sensory Testing, Pain Pressure Threshold, Temporal Summation, Conditioned Pain Modulation, and pain diary were assessed at inclusion and after 8 weeks. Proteomic analysis was performed on SF and blood samples before and after 8 weeks of treatment.

**Results:**

Linear combinations of serum or synovial biomarkers changed significantly alongside the effect measures and PainDetect scores following gold micro-particle treatment. Of particular interest was identifying multiple members of a molecular complex that is suggestive of neural tissue regeneration and modulation following gold micro-particle treatment.

**Conclusions:**

Gold microparticles are a possible future option for the treatment of knee osteoarthritis. The treatment triggers putative regenerative and inflammation-modulating molecular mechanisms.

## Graphical abstract

**Figure:**
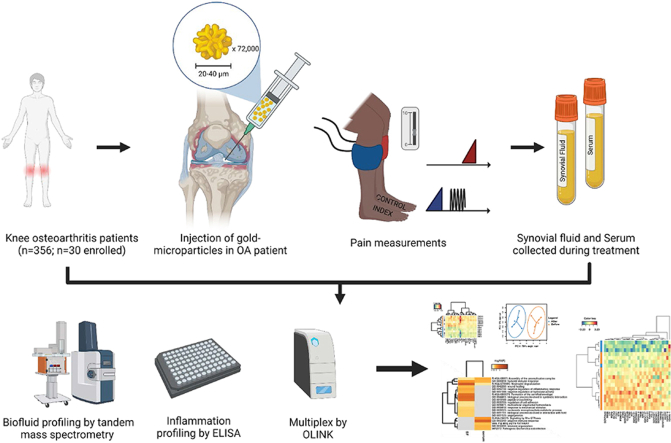

## 1 Introduction

Globally 595 million individuals are affected by osteoarthritis [1]. Compared to 2020, cases of osteoarthritis are expected to increase by 75% for knees before 2025. Knee osteoarthritis (KOA) is among the highest global causes of pain [2] and is responsible for substantial health and societal costs [3]. Treatment of osteoarthritis includes exercise programs, dietary weight management, topical NSAIDs, oral NSAIDs depending on cardiovascular comorbidities and tolerability, and intraarticular (IA) injections with corticosteroids, hyaluronic acid, or platelet-rich plasma [4]. IA treatment with corticosteroids and hyaluronic acid is equally effective and conditionally recommended with limited benefit beyond 3–4 weeks [3,4].

The IA corticosteroid injections are generally recommended for OA management and have relatively minor adverse effects. Other IA treatments are more controversial. Whilst corticosteroids continue to be recommended in international guidelines there is an acceptance that their use should be discouraged due to limited benefit beyond 3–4 weeks and an observed increase in OA progression with repetitive use [4,5]. Viscosupplementation demonstrates unpredictable improvement, and a recent systematic review does not support its broad use [6]. There is a lack of evidence to definitively recommend platelet-rich plasma for or against its use [7].

Metallic gold is recently proven soluble in the human body and gold ions bio-released from the metallic gold have an anti-inflammatory effect [[8], [9], [10]]. The causative mechanism is a macrophage-induced release of gold ions that influence the intracellular microenvironment and affect the immune and inflammatory response by changes in protein folding [[8], [9], [10]]. In our previous study, we found that gold microparticles had reduced knee osteoarthritic pain at two-year follow-up follow-ups and found significant proteomic changes in serum and synovial fluid (SF) within eight weeks [10]. We found multiple changes in inflammatory, and immunologic biomarkers. However, the relation between the different biomarkers and the outcome measures is unknown. We hypothesized that improvement in pain and function were associated with specific groups of biomarkers.

This study aimed to identify serum and synovial proteomic biomarkers that were associated with improvements in patient-reported outcomes and improvements in nociceptive and neuropathic pain following IA injection of gold microparticles in painful KOA.

## 2 Materials and methods

### 2.1 Study design, patient treatment, and ethical concerns

**Fig. 1:**
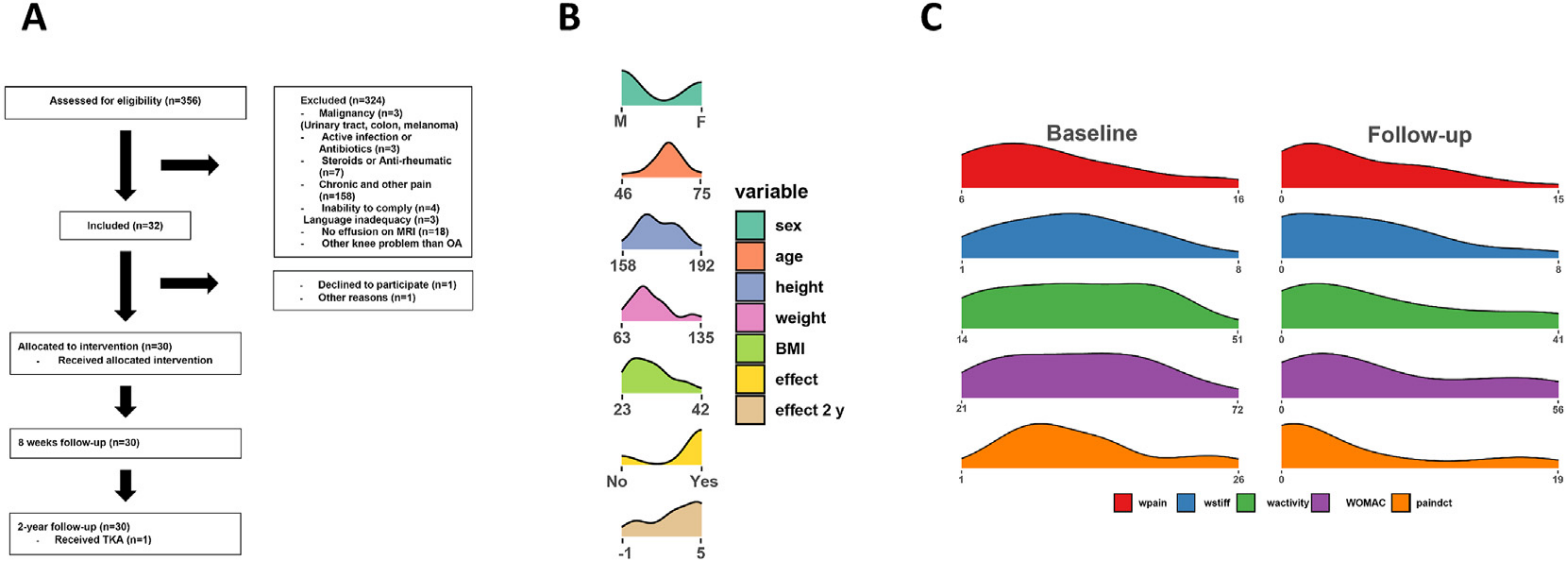
Study flowchart and treatment outcome received by patients at enrolment and follow-up (8 weeks) after initiation of nano-gold particle treatment. (A) The study flowchart gives an overview of the total number of patients (n ​= ​30) enrolled, excluded, and included for analyses (adapted from Ref. [10] with permission). (B) Patients who met the inclusion criteria are categorized by clinical measures across the cohort at inclusion (C) Clinical measures at inclusion and 8-week follow-up.

This is an ancillary analysis of a pilot trial investigating the effect of intraarticular gold microparticles on knee osteoarthritis [10]. In the original study, from January 2017, through March, 30 patients with radiographically confirmed moderate KOA (Kellgren-Lawrence grade ≥2), pain for more than 3 months, and maximal pain intensity VAS (Visual Analogue Scale, 0–10) ​≥ ​5 during the last week, and knee joint effusion on MRI that can be aspirated were enrolled at the Department of Orthopedic Surgery, Aalborg University Hospital, Denmark (Fig. 1A) [10]. The exclusion criteria were 1) malignancy, 2) active infection and antibiotic treatment, 3) active treatment with steroids, biological or other anti-rheumatic medication, 4) history of chronic pain condition, 5) inability to comply with the protocol, and 6) inadequacy in written and spoken national language.

The patients received gold microparticles, 20 ​mg sterile 99.99 %, a total of 72.000 particles, 20–40 ​μm in diameter (BerlockMicroImplants (BMI), Berlock ApS) [10,11] injections into the knee joint using the patient's own SF as the carrier. Specifically, 2 ​mL of SF aspirated from the knee, was mixed with the sterile gold microparticles, and re-injected into the patient's knee.

Blood and synovial fluid samples were collected at the day of administration and at the day for the 8-week follow-up. The synovial fluid used for analysis were collected before the injection of the gold microparticles using 2 ​mL of the collected synovial fluid collected as the carrier. The same puncture needle was used for both collection of the synovial fluid and the injection of the treatment. The blood samples (6 ​mL) were centrifuged at 3000 RPM for 15 ​min and pipetted into two cryotubes. The synovial fluid samples (4–6 ​mL) were centrifuged at 2200 RPM for 10 ​min and pipetted into cryotubes. The storage of samples was at −80 ​°C and in multiple aliquots. All the samples were analyzed simultaneously after completion of the project.

At the initial evaluation and again at 8 weeks, we conducted a comprehensive clinical assessment that included collecting clinical outcomes and serum and SF samples. Clinical outcome measures were once again collected at a two-year follow-up to assess the long-term effects of treatment. Between 1 and 2 years after treatment 1 patient with the Kellgren-Lawrence score IV received total knee arthroplasty, and 1 patient needed an arthroscopic meniscectomy after an injury.

The Consort guideline for reporting non-randomized pilot and feasibility studies was followed [12]. All authors take responsibility for the integrity and accuracy of the reported data and the credibility of the study to the protocol. The protocol is available at https://vbn.aau.dk/da/projects/gold-microparticles-for-knee-osteoarthritis.

### 2.2 Primary and secondary outcome measures

The main patient-reported outcome measure pre-treatment and at follow-up was the Western Ontario and McMaster Universities Arthritis Index (WOMAC) sub-scores for pain, stiffness, and function, containing 24 questions: 5 pain questions, two stiffness questions, and 17 physical function questions. Each question utilizes a 5-point scale, from 0 (none) to 4 (extreme) [13].

Using the Global Rating of Change Scale [14] we asked the question, *“Concerning your knee, how will you describe yourself compared to immediately before the injection of gold into your knee?”* and evaluated the answer on an 11-point scale from very much worse (−5) to complete recovered (5) with a score of “0” indicating no changes. Patients achieving a score >0 were considered responders and a score <1, were non-responders. The secondary outcome measure PainDetect questionnaire [15] comprises three major components, gradation of pain, pain course pattern, and radiating pain. Seven questions evaluate the gradation of pain. The patient scored each question using a 0 to 5 score with 0 ​= ​never, 1 ​= ​hardly noticed, 2 ​= ​slightly, 3 ​= ​moderately, 4 ​= ​strongly and 5 ​= ​very strongly. There is one question evaluating pain course patterns. Patients select from one of four pictures indicating which pattern best describes their course of pain. Each picture is associated with a unique score of 0, -1, or +1 (2 pictures have this score possible). There is one question evaluating radiating pain with a yes (score of +2) or no (score of 0) response option. PainDetect questionnaire is scored from 0 to 38, with total scores <13 considered to represent nociceptive pain, 13–18 possible neuropathic pain, and >18 representing neuropathic pain.

### 2.3 Statistical analysis

The raw diaPASEF files were processed with Spectronaut™ powered by Pulsar (version 14.10.201222.47784; Biognosys) (Supplemental material). Standard default settings were employed, including QUANT2.0 label-free quantitative using the MS2 profile. Normalized retention time-based liquid chromatography run alignment were applied using the internal peptide library hosted by Spectronaut™. All label-free quantitative data were normalized on the global medial and filtered by a q-value of 0.01 (equal to an FDR of 1%). The data was then preprocessed in Perseus, including log2 transformation, averaging of technical duplicates by calculating the mean, and imputation of missing values from a normal distribution to simulate signals from low-abundance proteins. Furthermore, keratin contaminants originating from the sample preparation were removed.

A principal component analysis (PCA) was made to visualize the major sources of variation in the SF and serum datasets. The PCA was conducted using the mixOmics R package, which can handle repeated measurement experiments by performing within-subjects matrix decomposition internally, thereby focusing on the variation within each individual across baseline and follow-up.

To explore relationships between proteins and clinical outcomes, we integrated data using the sparse Partial Least Squares (sPLS) function from the mixOmics R package. The model was executed in regression mode, incorporating multilevel decomposition to account for repeated measures. Default settings were used, including a tolerance level of 1e-06 during the iterative process, a maximum of 100 iterations, and standardization of each block to zero mean and unit variance.

The optimal number of features to retain (ranging from 1 to 25) was determined using the associated tune.splslevel function for repeated measurements. This tuning criterion was based on maximizing the Pearson correlation between the components of the proteome data sets and each of the outcome measures (i.e., WOMAC Function, Pain, or Stiffness, and PainDetect score).

Data Integration Analysis for Biomarker discovery using Latent cOmponents (DIABLO) was made using the block.splsda function from the mixOmics R package. This was to identify associated SF and serum proteins that could explain the difference between responders and non-responders at 8-week follow-up. The model was executed using default settings, including a including a tolerance level of 1e-06 during the iterative process, a maximum of 100 iterations, and standardization of each block to zero mean and unit variance. The optimal number of features to retain (ranging from 5 to 10) on each component was determined using the tune.block.splsda function. This involved using five-fold internal cross-validation, that was repeated 50 times, and the performance of the model was evaluated based on classification (balanced) error rate, with the mahalanobis distance serving as the distance metric to estimate the classification error rate.

A sPLS discriminant analysis (sPLS-DA) model was likewise made using the splsda function from the mixOmics R package. This was to identify associated SF proteins that could explain the difference between responders and non-responders at 8-week follow-up, without considering their relationship with serum proteins. The splsda model was executed using default settings, including a including a tolerance level of 1e-06 during the iterative process, a maximum of 100 iterations, and standardization of each block to zero mean and unit variance. The optimal number of features to retain (ranging from 1 to 30) on each component was determined using the tune.splsda function. This involved using three-fold internal cross-validation, that was repeated 50 times, and the performance of the model was evaluated based on classification (balanced) error rate, with the maximum distance serving as the distance metric to estimate the classification error rate. Due to the limited number of subjects, the evaluation of DIABLO and sPLS-DA model performance did not involve splitting the data into separate training and test sets which represents a noteworthy limitation.

Sample plots, heatmaps with hierarchical clustering (Euclidean distance, complete-linkage), circos plots, and loading plots were made using the mixOmics functions plotIndiv, cim, circosPlot, and plotLoadings, respectively.

Finally, to assess the biological relevance of the proteins that were covarying with treatment outcomes, a functional enrichment analysis was made in Metascape [16]. All over and under-represented data from SF and serum were used as input in Multiple gene lists analysis. For each given gene list, pathway and process enrichment analysis have been carried out in Metascape from the following ontology sources: KEGG Pathway, GO Biological Processes, Reactome Gene Sets, Canonical Pathways, CORUM, WikiPathways, and PANTHER Pathway. In Metascape all genes in the genome were used in the enrichment background. Terms with a p-value <0.01, a minimum count of 3, and an enrichment factor >1.5 (the enrichment factor is the ratio between the observed counts and the counts expected by chance) are collected and grouped into clusters based on their membership similarities. More specifically, p-values are calculated based on the cumulative hypergeometric distribution, and q-values are calculated using the Benjamini-Hochberg procedure to account for multiple testings.

## 3 Results

### 3.1 Treatment of KOA patients with intraarticular gold microparticles improves short and long-term outcomes

Cohort characteristics of 30 patients in the OA GOLD study set have been described previously [10] (Table 1, and Fig. 1A). The OA GOLD cohort was generated by restrictive inclusion criteria and the analytical data set was generated by integrated analysis of blood and SF samples and patient-reported outcome measures. (Supplemental data S1–S6). All 30 patients completed the follow-up. No adverse or serious events were recorded. For all objectives, all 30 patients were included in the analysis.

**Table 1:** Baseline characteristics of the 30 patients. Values are median and range; and mean and 95% CI. Scores on the Kellgren–Lawrence scale range from 0 to 4, with a score of 2, 3, or 4 indicating definite osteoarthritis and higher scores indicating more severe disease.

Female/Male sex	12/20
Age – year	63 (46–75); 62 (49.5–74.2)
Body mass index	28.8 (22.8–41.7); 29.5 (10.7–39.3)
Clinical sign of effusion	3/30
Volume (ml) synovial fluid aspirated	6 (−0.68-12.9); 8 (3–18)
Kellgren-Lawrence Score	
II	3
III	28
IV	1
Womac Scores	
Pain	9 (6–16); 9.3 (5.1–3.2)
Stiffness	8 (1–4); 3.8 (−0.27-7.3)
Function	29 (51–14); 29.5 (9.5–49.5)
Pain evaluation	
Pain Detect	10 (1–26); 11.3 (−1.2-23.8)
Pressure Pain Threshold (kPa)	598 (276–1043); 572 (380–764)
Temporal Summation of Pain (% VAS)	103 (−69.2–694); 129 (-192-450)
Conditioned Modulation of Pain (kPa)	−3.45 (−56.6–125); 8.38 (−77.7–94.4)

To further investigate the treatment outcome, we plotted the patient clinical measures and distribution hereof (Fig. 1B). The WOMAC-Function, WOMAC-stiffness, WOMAC-pain, PainDetect, and Kellgren-Lawrence scores were assessed at 8 weeks (mean 8.2 weeks) and at two years follow-up (mean 25.1 months). All clinical outcome measures were observed visually and statistically to improve from baseline to 8-week follow-up (Fig. 1C; Supplemental Table S1). For the following sections, we applied a statistical workflow optimized for delineating proteomics-based outcomes.

**Fig. 2:**
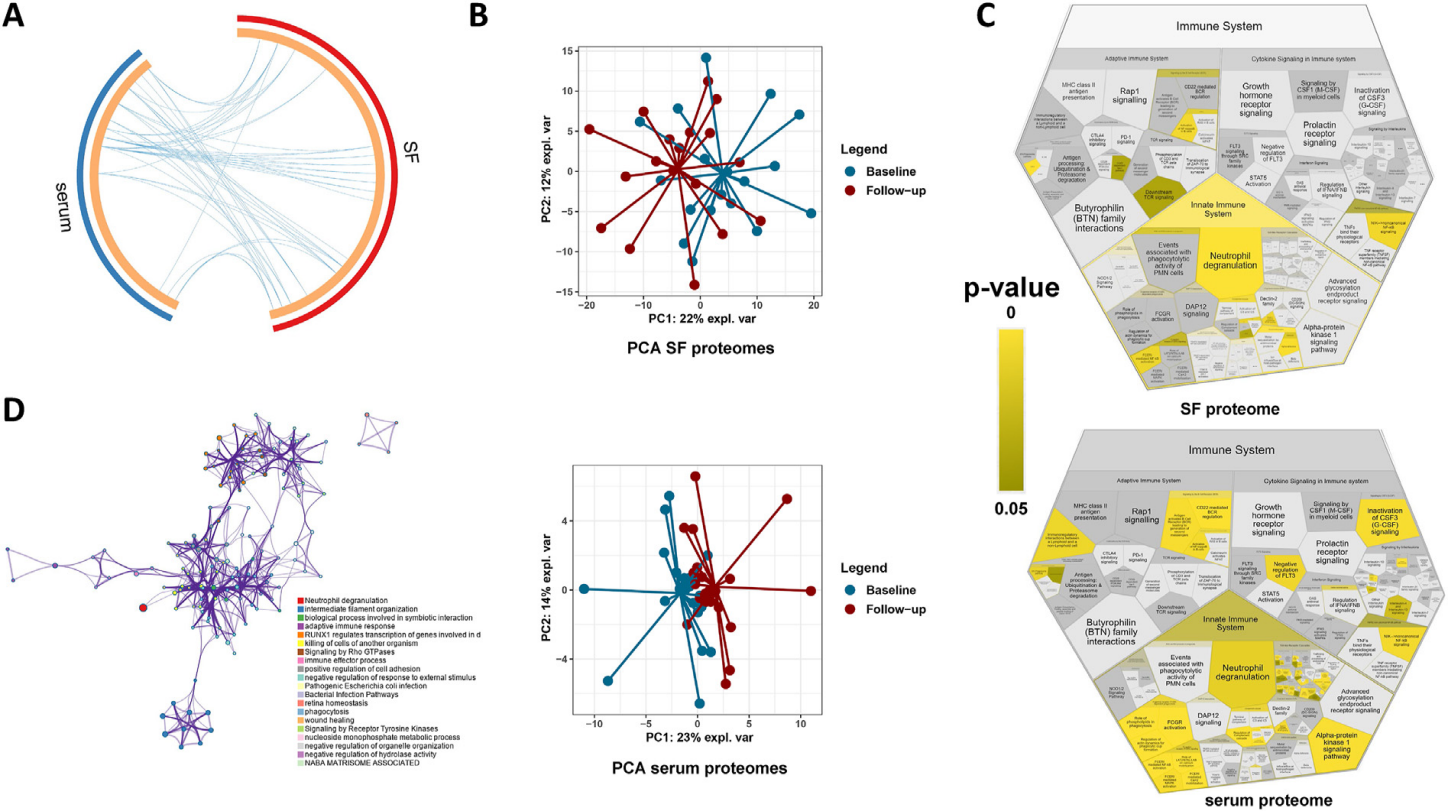
Proteomic analysis and inflammation profiling of SF and serum. (A) Overlap of over- and underabundant proteins in respective biofluids visualized by a Circos plot illustrating the overlap between gene lists where blue curves link identical proteins. (B upper and lower) PCA of SF and serum proteins indicates a shift in proteome between baseline and 8-week follow-up. (C) Proteome enrichment analysis of SF and serum over and under-represented proteins show proteomic changes in multiple immune system-related processes in each biofluid. (D) Proteome enrichment analysis of SF and serum over and under-represented proteins with distinct clusters of molecular functions locally (SF) and systemically (serum).

Our previous findings investigating the inflammatory profiles of enrolled patients indicated both localized and systemic regenerative and immunomodulatory processes in the treated knee joint and to a lesser degree, systematically in serum [10]. Here a total of 39 SF-associated proteins and 32 serum proteins were found significantly over- or underabundant from *a total of 529 and 380 quantifiable proteins, respectively*. In a secondary explorative analysis, when comparing the two sets of associated proteins, a large overlap can be observed (Fig. 2A). For each biofluid, an unsupervised PCA allowed partial separation of each biofluid when comparing patients at inclusion and at 8-week follow-up (Fig. 2B upper and lower). Thus, SF and serum profiles reflect a change in local (SF) and systemic (serum) proteomes associated with the gold microparticle treatment. The significantly regulated SF proteins from baseline to 8-week follow-up are mainly associated to overrepresented cellular processes related to inflammation reducing innate immune system protein regulations with FDR 2.29 ​E^−7^ (SERPINB3, APCS, FCN3, CALML5, DCD, KRT1, DEFA3, DEFA1, MPO, PSMA5, CDC42, PNP, FABP5, PSMC3, RAC2, CTSG, RAC1, LTA4H, PSMF1, PGLYRP1, IGHV3-7, ELANE) and neutrophil degranulation with FDR 2.73 ​E^−7^ (SERPINB3, CALML5, KRT1, DEFA1, MPO, PSMA5, PNP, FABP5, PSMC3, CTSG, RAC1, LTA4H, PGLYRP1, ELANE) [10]. Further of investigation, the molecular and cellular mechanisms associated with these proteins identified confirmed both common and distinct immunological changes in the above categories (Fig. 2C upper and lower; 2D).

### 3.2 WOMAC index components pain, stiffness, function and PainDetect co-varies with proteins related to the innate immune system, protein complexes, and complement system

We initially applied sPLS as an unsupervised model to identify which SF and serum proteins were associated with KOA outcome measures, including WOMAC pain, WOMAC stiffness, and WOMAC function [13] as well as PainDetect scores [15]. The sPLS achieves this by identifying a linear combination of SF and serum proteins that covary with the aforementioned clinical outcome measures.

**Fig. 3:**
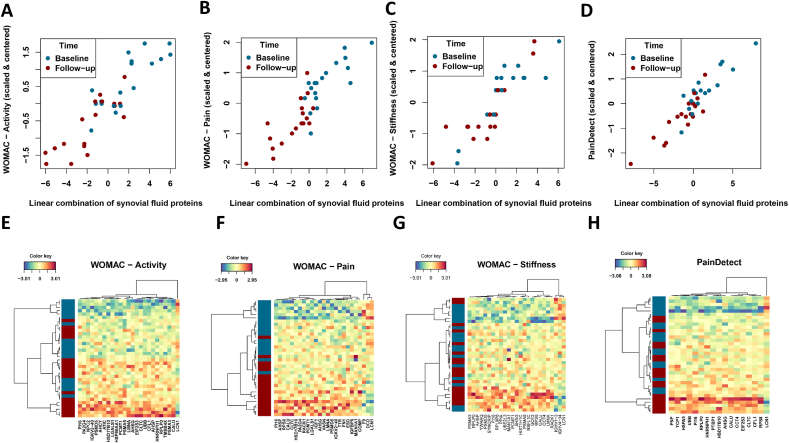
sPLS score plots showing distribution of patients based on of the linear combination of SF proteins (component 1 score - x-axis) that covaried with (A) WOMAC – function; (B) WOMAC – pain; (C) WOMAC – stiffness and (D) PainDetect. The Y-axis represents centered and scaled clinical outcome measures. Figures E–H are heatmaps showing the clustering of proteins based on the SF proteins that were associated with (A) WOMAC – function; (B) WOMAC – pain; (C) WOMAC – stiffness and (D) PainDetect.

The Pearson correlation coefficient between the WOMAC scores (function, pain, stiffness) (Fig. 3A–D) and the linear combination of the selected SF proteins which was found to be r(34) ​= ​0.89, 0.88, 0.88, p ​< ​10^−14^; respectively (Fig. 3A–C). The Pearson correlation coefficient between the PainDetect score and the linear combination of 17 selected SF proteins was found to be r(34) ​= ​0.92, p ​< ​10^−14^. The linear combination of SF proteins and the PainDetect score partly explains the differences between baseline and follow-up samples (Fig. 3D). When comparing the associated protein variables, a high degree of overlap was observed (Fig. 5A and B).

The functional enrichment analysis revealed that the SF proteins were associated with 1) gene and protein expression by JAK-STAT signaling after interleukin-12 stimulation, 2) metabolism of RNA and axon guidance, 3) signaling by FGFR2, 4) platelet/neutrophil degranulation, 5) negative regulation of peptidase function and proteolysis, 6) transport of small molecules and 7) negative regulation of cell differentiation (Fig. 5C). The Molecular Complex Detection algorithm incorporated in Metascape [17] identified a subset of nine proteins known to physically interact with each other in a functional protein complex (CFL1, CLTC, EIF2S3, HSD17B10, HSPA9, PTBP1, RPS8, UBB, CCT4). The most significantly enriched biological processes among these proteins were 1) axon guidance (p ​= ​0.000013), 2) nervous system development (p ​= ​0.000016) and 3) metabolism of RNA (p ​= ​0.000032). Furthermore, eight of the nine proteins have been associated to exosomes in blood (p ​= ​0.00028).

**Fig. 4:**
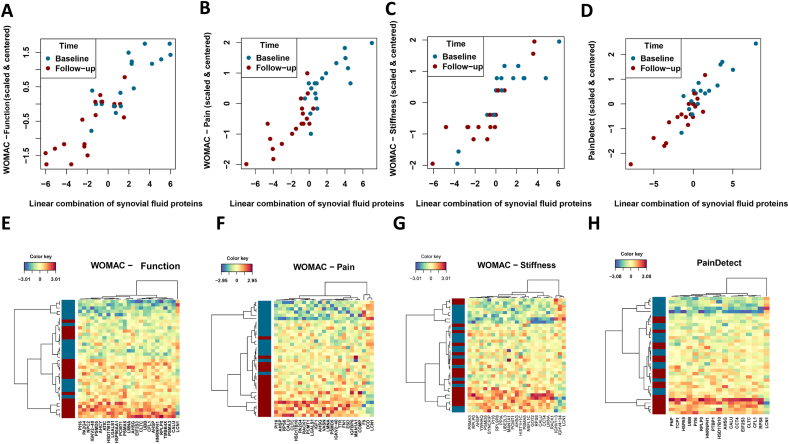
sPLS score plots showing distribution of patients based on of the linear combination of serum proteins (component 1 score - x-axis) that covaried with (A) WOMAC – function; (B) WOMAC – pain; (C) WOMAC – stiffness; and (D) PainDetect. The Y-axis represents centered and scaled clinical outcome measures. Figures E–H are heatmaps showing the clustering of proteins based on the serum proteins that were associated with (A) WOMAC – function; (B) WOMAC – pain; (C) WOMAC – stiffness; and (D) PainDetect.

**Fig. 5:**
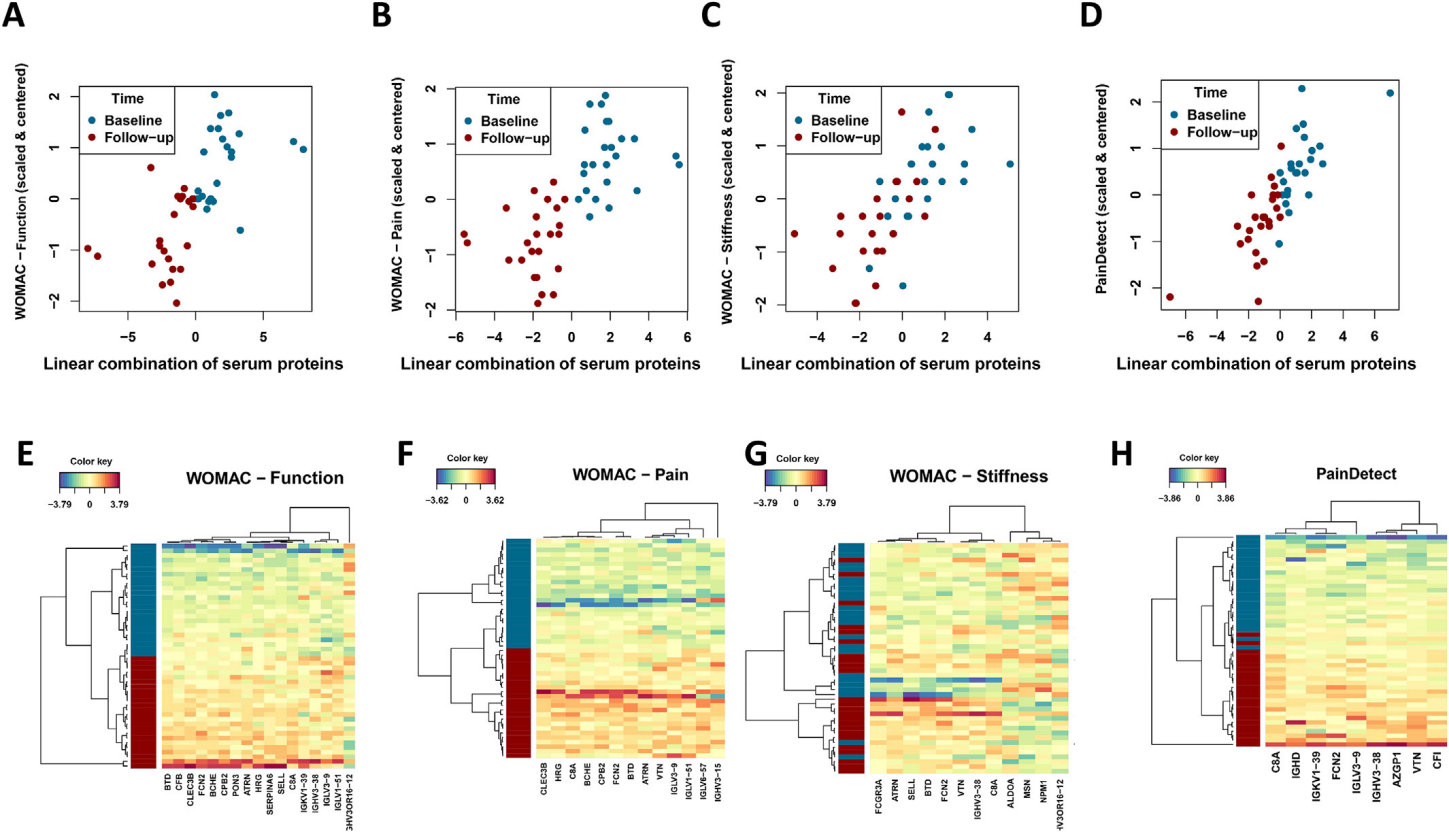
Proteomic overlap with clinical scores and associated biomarkers (A) Venn diagram of overlapping proteins associated with clinical measures in SF; (B) Circos plot illustrating overlap between gene lists where purple curves link identical proteins. SF proteins that span multiple lists are colored in dark orange, and proteins unique to one list are shown in light orange; (C) Heatmap of enriched GO biological processes across input SF proteins, colored by p-values (D) Venn diagram of overlapping proteins associated to clinical measures in serum; (D) Circos plot illustrating overlap between gene lists where purple curves link identical proteins. Serum proteins that span multiple lists are colored in dark orange, and proteins unique to a single list are shown in light orange; (F) Heatmap of enriched GO biological processes across input serum proteins, colored by p-values.

Next, we calculated the Pearson correlation coefficient between the WOMAC scores (function, pain, stiffness) and the linear combination of 12–17 selected serum proteins to be r(34) ​= ​0.65, 0.67, 0.68, p ​< ​10^−14^, respectively (Fig. 4A–C). The Pearson correlation coefficient between the PainDetect score and the linear combination of 9 selected serum proteins was r(34) ​= ​0.79, p ​< ​10^−14^. The linear combination of serum proteins and the PainDetect score partly explains the differences between baseline and follow-up samples (Fig. 4D). When comparing the associated protein variables, a high degree of overlap can be observed (Fig. 5B–E).

The functional enrichment analysis revealed that the seven proteins were associated with multiple immune response-related biological processes including complement cascade and regulation hereof (Fig. 5D and E). The most significantly enriched biological processes among these proteins were 1) Negative regulation of fibrinolysis (p ​= ​0.0046), 2) Negative regulation of blood coagulation (p ​= ​0.0250) and 3) Complement activation (p ​= ​0.0357).

### 3.3 Co-variation analysis of SF and serum identifies extracellular matrix turnover as key factor for positive treatment outcome

**Fig. 6:**
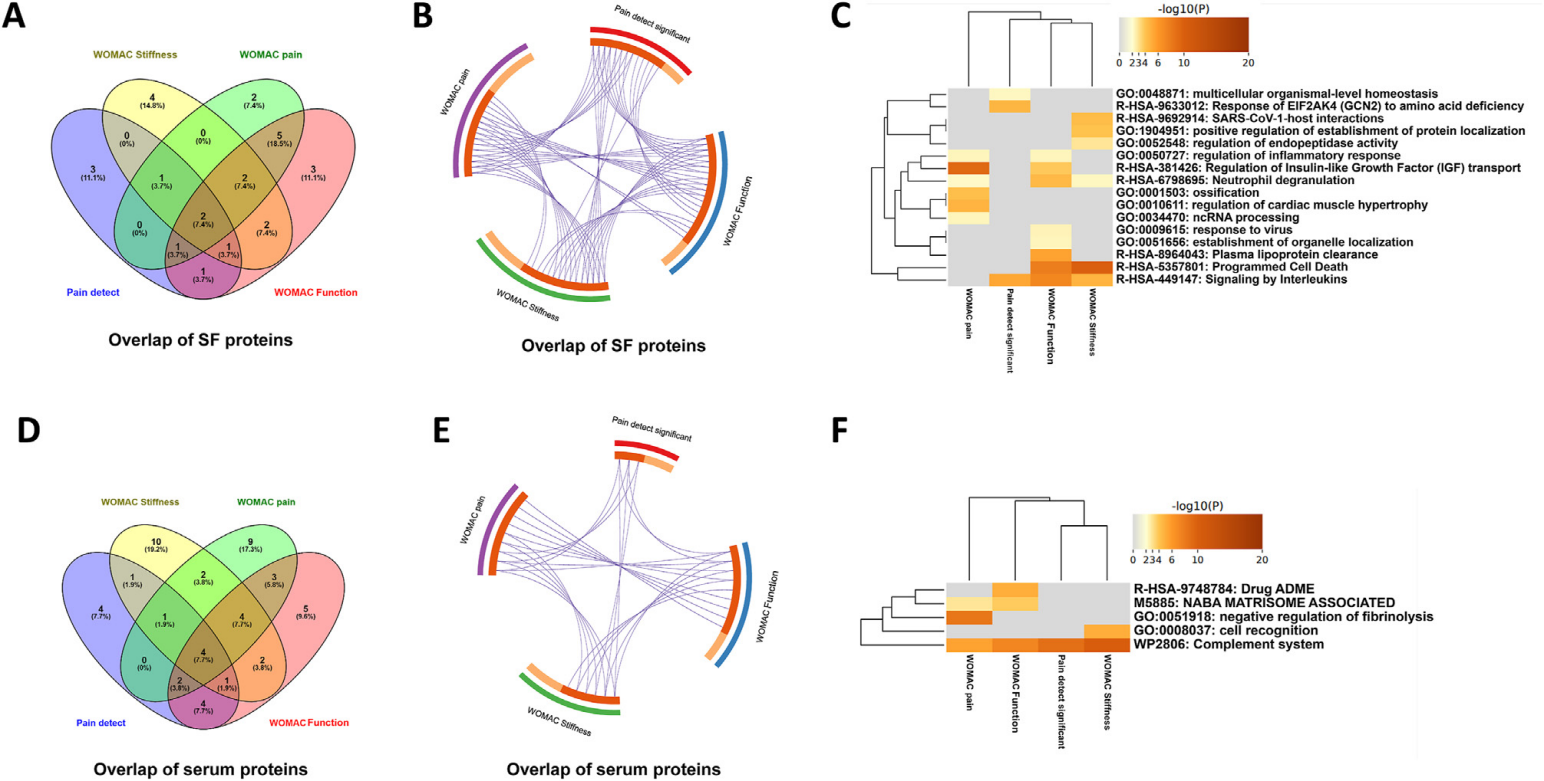
Analysis of systemic and local inflammation to treatment effect scores. (A) The DIABLO analysis integrating SF and serum proteins was able to identify protein biomarkers for separation of proteins according to their 8-week treatment response, (B) Heatmap of the SF and serum proteins that were associated with 8-week treatment response (0,1), (C) Circos plot illustrating correlations (>±0.7) between SF and serum proteins.

To investigate the protein variables in SF and serum that co-vary with treatment outcome measures at 8 weeks we applied a supervised statistical approach termed DIABLO, a multi-omics integrative method that seeks common information across different data types through the selection of a subset of molecular features, while discriminating between multiple phenotypic groups [18]. Specifically, DIABLO was used to identify the most highly associated variables across the SF and serum proteomes that could simultaneously discriminate between patients based on their 8-week treatment response (No effect versus Effect) (Fig. 6A). A total of fifteen proteins were found to be associated with the 8-week treatment response (Fig. 6B). Nine of these were SF proteins, namely: CTSD, PSMD10, SERPINB3, S100A9, SBSN, IGHV5−51, SERPINA4, CLEC3B, and SERPINF1 (Fig. 6B). The remaining six proteins that were associated with the 8-week treatment response were the serum proteins: VWF, LBP, FCGBP, IGHV1OR15−1, APOA1, and DBH (Fig. 6 B).

When applying a correlation cutoff at 0.7, two serum proteins, namely LBP and FCGBP, were found to be negatively correlated with the SF proteins, and these had a higher relative abundance in patients with a worse (=0) 8-week treatment response (Fig. 6C).

The biological function of the fifteen proteins was found to be associated with extracellular matrix components (p-values 0.0046–2.55e^−06^) and proteolytic degradation hereof (p-value 0.0011) (Fig. 6D).

### 3.4 Treatment effects can be associated with ECM turnover and neutrophil degranulation processes

**Fig. 7:**
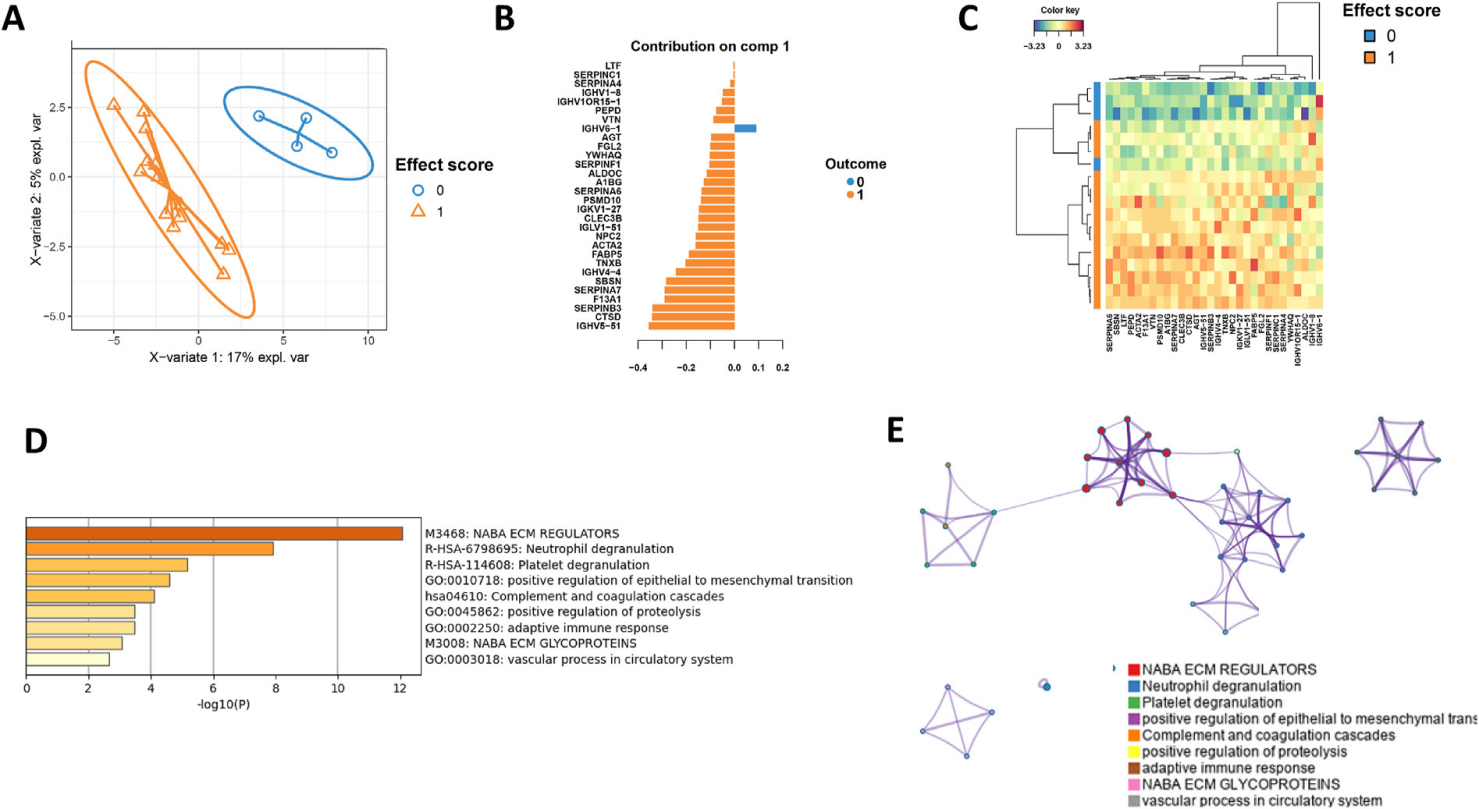
sPLS-DA analysis for identification of proteins that could discriminate between patients based on their 8-week treatment response. (A) Score plot indicating full separation of well-responders and non-responders based on effect score; (B) The 29 proteins with the highest predictive performance for discrimination of well-responders and non-responders (orange color); (C) Heatmap showing how patients clustered based on the selected 29 proteins; (D) Biological function of the 29 SF proteins included extracellular matrix processing and immune responses including neutrophil degranulation; (E) A network presentation of the biological pathways that were associated with 8-week treatment response to gold microparticle injection for treatment of KOA.

We finally employed the supervised sPLS-DA analysis to identify SF proteins for discrimination of patients based on their 8-week treatment response (Fig. 7A). The sPLS-DA was able to discriminate between patient groups on component 1 (Fig. 7A) and the proteins responsible for doing so are shown in Fig. 7 B–C. Functional enrichment analysis of these proteins revealed that the 29 proteins were associated with 1) neutrophil degranulation, 2) regulation of IGF transport and uptake by IGF binding proteins and platelet degranulation, 3) plasma lipoprotein clearance and multiple intracellular signaling pathways related to cell cycle and apoptosis among others, 4) protein stabilization and cell growth, 5) EPH-Ephrin signaling and axon guidance, 6) negative regulation of peptidase activity and proteolysis, 7) signaling by receptor tyrosine kinases and ossification and 8) cell-cell adhesion.

## 4 Discussion

We identified linear combinations of serum or synovial fluid biomarkers that changed significantly alongside the clinical outcome measures of IA gold microparticle treatment for KOA. Our findings show a range of identical over- and underabundant proteins in each biofluid investigated. Overall, the serum and SF proteomes shifted during 8 weeks post-treatment finding that over and under-represented proteins were associated with a reduction in inflammatory signaling processes and mechanisms including innate immune system-associated processes including neutrophil degranulation and regulation through nuclear factor-κB signaling. The understanding of the interactions of the immune system with biomaterials and how immune cells participate in the process of wound healing and regenerative processes are critical for the development of successful solutions such as gold microparticle use in painful KOA. The role of neutrophils in the inflammatory response has gained increased interest over the last years and has recently been reviewed in facilitating biomaterial–tissue integration and ultimately in tissue repair/regeneration [19]. Here Sousa et al. summarized the immunomodulatory actions were attributed to positive and negative effects. Among positive mechanisms tissue repair have been reported in terms of anti-inflammatory and pro-regenerative actions by upregulating the expression of important cytokines and chemokines such as tumor necrosis factor (TNF)-α, IL-1β, IL-6 and MCP-1 enabling recruitment of immune cells important for the wound healing processes. Tissue remodeling via neutrophil release of urokinase-type plasminogen activator or elastase, heparinase, and cathepsin could promote beneficial ECM turnover. On the other hand, prolonged neutrophil activity was found to impair tissue repair/regeneration at elevated levels of ROS, through degranulation they release toxic mediators or prolonged activity MMPs. We speculate that if micro gold implants increase the longevity of neutrophil signaling significantly, their role in the inflammatory and wound healing processes could be more relevant than previously considered [20].

Our unsupervised sPLS analysis integrating all proteomic protein variables associated also the above mechanisms to the individual scores of WOMAC (function, pain, stiffness) including regulation of ECM proteolysis and turnover from baseline to 8 weeks follow-up [10]. The SF biomarkers were additionally associated with multiple biological processes that are collectively suggestive of tissue regeneration and nervous system remodeling following gold microparticle treatment.

We identified linear combinations of serum or synovial biomarkers that change significantly alongside PainDetect scores following gold microparticle treatment for KOA. Both the serum and SF biomarkers were associated with immune-related processes. The SF biomarkers were also associated with other biological processes, but of particular interest was identifying multiple members of a molecular complex that is suggestive of neural tissue regeneration and modulation following gold micro-particle treatment. The study further demonstrates the feasibility of utilizing protein biomarker signatures in future clinical decision-making.

Neutrophils drive a transiently intensified anti-inflammatory response that is protective against the transition from acute to chronic pain [21]. Data shows that an anti-inflammatory immune response is driven by neutrophils early after injury and facilitates pain resolution. Specifically, degranulation pathways driven by neutrophil activation displayed the largest changes in expression.

Our findings of co-varying proteins from baseline to positive treatment outcome enable a catalog of biomarkers for effective treatment outcomes applicable for companion diagnostic approaches [22]. Proteomic analysis of IA treatment for OA in animal studies indicates induced chondrogenesis of mesenchymal stem cells [23], modulation of inflammatory OA pathogenesis by nanozymes [24], and attenuation of OA by zinc finger proteins [25]. Some studies suggest there is an antioxidant property of gold-based therapies that protects the cartilage structure [[26], [27], [28]]. These studies' proteomic analysis supports the proteomic findings in this study as well as the findings in our previous study [10].

Earlier publications have identified differences in protein profiles between OA and non-OA SF and a tissue-dependent release of proteins in human knee OA [29] following the differences we have found in our study before and after treatment with IA gold microparticles.

A general study limitation is the use of highly selected patients with knee OA that hamper the generalization of the findings in this study. In addition, the changes measured may be due to a natural course of the disease, the intention to treat, or the puncture and aspiration by itself. The study was not blinded including no control group and may overestimate the effect. The multiple statistical tests performed increase the false-positive error rate and may additionally overestimate the effect. It may not be surprising compared to our previous paper [10] that we find the associations of pain and function to specific biomarkers. However, the changes in identified biomarkers we find in OA-related alterations in the proteomic composition of both SF and serum related to patient-reported outcomes have the potential to improve patient care by facilitating specific future IA therapeutic interventions.

It is a strength that synovial fluid and blood samples were collected at the day of the treatment and again at the day of the 8-week follow-up. Another strength is that all samples were stored at −80 ​°C in multiple aliquots and analyzed simultaneously after completion of the project. A weakness is the storage for up to 5 years in this study can lead to altered concentration and may be safe for up to seven years [30].

## 5 Conclusion

We identified linear combinations of serum or synovial biomarkers that changed significantly alongside the clinical outcomemeasures of gold micro-particle treatment for KOA. Both the serum and synovial fluid biomarkers were associated with acute and early inflammatory processes and regulation of proteolysis of extracellular matrix and regenerative processes. The synovial fluid biomarkers were additionally associated with multiple biological processes that are collectively suggestive of tissue regeneration and nervous system remodeling following gold micro-particle treatment. The study further demonstrates the feasibility of utilizing protein biomarker signatures in future validation studies.

## Data sharing

All data, code, and materials used in the analysis are available upon request for the purposes of reproducing or extending the analysis via the corresponding author, in accordance with local and institutional guidance and legal requirements. The proteomics data have been deposited to the ProteomeXchange Consortium via the PRIDE partner repository with the dataset identifier PXD030746.

## Trial registration

The study followed the principles of the Declaration of Helsinki and was approved by the local ethics committee of the North Denmark Region by July 27, 2016 (N-20160045). The regional data protection agency approved the project by July 06, 2016 (2008-58-0028, ID 2016–116) and registered in ClinicalTrial.Gov by January 04, 2018 (NCT03389906).

## Credit author statement

SR was the principal investigator in the study and participated in data collection, analysis, and interpretation. SR and AS wrote the draft manuscript. AS, JS, and CA performed proteomics profiling inflammation analysis, and bioinformatics investigation. All authors assisted in analyzing and interpreting the data and contributed to writing the manuscript. All authors read and approved the final manuscript. All authors are responsible and account for the work's originality, accuracy, and integrity. AI and AI-assisted technology were not used.

## Funding

Department of Clinical Medicine provided funding for this study. Center for Neuroplasticity and Pain supported this study by the Danish National Research Foundation (DNRF121) and the Danish Rheumatism Association (R204-A7645). The Danish National Mass Spectrometry Platform for Functional Proteomics (PRO-MS; grant no. 5072-00007B); The Obelske family foundation, the Svend Andersen Foundation, and the SparNord foundation are acknowledged for grants to the analytical platform, enabling parts of this study.

## Declaration of competing interest

All authors state that no conflict of interest exists.
