# Supplementary material for: Intraarticular gold for knee osteoarthritis: An ancillary analysis of biomarkers and outcome of a pilot study
Source: Osteoarthr Cartil Open. 2024 Aug 31;6(4):100514. doi: 10.1016/j.ocarto.2024.100514 (PMC11406078; doi:10.1016/j.ocarto.2024.100514)
Supplement: Multimedia component 1 [file mmc1.docx]

**Supplemental information**

***Detailed protocols***

***Trial registration:***

The regional data protection agency approved the project by July 6, 2016 (2008-58-0028, ID 2016-116). The local Ethics Committee of The North Denmark Region approved the project by July 28, 2016 (N-20160045), <http://www.nvk.dk/~/media/NVK/Om-NVK/Godkendte-projekter/2016/Q2-2016.pdf?la=da>. https://clinicaltrials.gov/ct2/show/NCT03389906, initial registration date January 4, 2018

***Liquid biopsy collection and biobanking***

SF from the knee was centrifugated for 10 minutes at 2200G to remove cell debris and the supernatant was frozen in aliquots at -80℃ for future analysis. The 6 ml venous whole blood sample was centrifugated at 3000 RPM for 15 minutes. Likewise, the serum fraction was stored for future analysis at -80℃. All sample data were collected and kept following Danish legislation on data handling at the Department of Health Science at Aalborg University Hospital. Each patient sample was divided into aliquots to avoid unnecessary freezing and thawing cycles.

***Immune profiling by olink Proximity Extension Assay***

An olink Proximity Extension Assay (PEA) was carried out on all OA synovial fluid samples to identify smaller and less abundant peptides affected by the gold treatment. This panel comprises 96 immune-related biomarkers common for many disease pathologies. Measurement of these 96 biomarkers was performed by Olink Bi-oscience analysis service (Uppsala, Sweden), using the Proseek® multiplex Inflammatory 96*96 kit. The Proseek® reagents are based on a PEA technology, which binds 96 oligonucleotide-labelled antibody probe pairs to the target biomarker. For further quantification, real-time PCR was performed. Olink wizard and GenEx software were used for further data analysis. Proseek® data are presented as arbitrary units (AU) on a log2 scale. Every marker was categorized by current literature in one or more categories. The abbreviations and full names of the 96 biomarkers and their categories are presented in Supplementary material online, Table S1.

***cfDNA as a marker of innate immunity***

SF and serum samples were thawed and centrifuged at 15,000g for 15 minutes, diluted 1:25 in 10 mM Tris, pH 8.0 with 1 mM EDTA (TE-buffer). The Quant-iT™ PicoGreen™ dsDNA Assay Kit (ThermoFisher Scientific) was used according to the manufacture’s instruction using 96 well Microplates, PP, F-Bottom black chimney well design (Sigma Aldrich). Four-fold dilution series of DNA were included on all plates (1µg/mL, 250ng/mL, 62ng/mL, 15,6ng/mL, 3,9ng/mL, 970pg/mL, 243pg/mL, 0pg/mL). Samples and standards were prepared and measured in duplicates. Plates were measured on an Enspire Multimode Plate Reader (Perkin Elmer, Waltham, MA) with excitation 480 nm and emission 520 nm. To enroll higher confidence in the following analysis all measurements were done in technical duplicates and only values varying less than 5% between these were used for further analysis.

***Filter aided sample preparation for proteomics profiling of SF and serum***

All samples were prepared for mass spectrometry with filter aided sample preparation (FASP). 100 ug protein (determined by BCA) was transferred to a spin filter tube (VWR North America Filter, #FC9847) with 200 ul 0.5% SDC in 50mM TEAB. The proteins were denatured in a heat block at 95°C for five minutes and centrifuged at 14000g (Hettich Zentrifugen, Rotina 380R) for another five minutes, the flow-through was discarded. This step was performed twice. Subsequently, the samples were reduced and alkylated by incubation with a mixture of digestion buffer, 10 mM TCEP and 50 mM chloroacetamide (CAA) for 30 minutes at 37°C. Samples were then cen-trifuged at 14000g for five minutes, followed by a digestion buffer wash and centrifugation, the flow through was discarded thoroughly by pipetting. The spin filters were incubated overnight at 37°C with trypsin (Thermo Scientific #815-968-0747) in digestion buffer. The tubes were centrifuged at 14000g for five minutes and rinsed with di-gestion buffer followed by centrifugation at 14000g for five minutes, leaving the di-gested proteins in the collection tubes. Remaining detergents were removed by phase separation with ethyl acetate (VWR, LOT#83621.320) and trifluoroacetic acid (TFA) (Thermo Fisher Scientific, LOT#a116-50). After adding the phase separation reagents to the samples, they were vortexed thoroughly and centrifuged for five minutes at 14000g which left an upper phase containing detergents and a lower phase containing digested proteins. The upper phase was carefully removed by pipetting. The phase separation was repeated with ethyl acetate but no TFA.

***Processing and data acquisition of biofluid samples by LC-MS/MS***

Peptides were reconstituted in 2% acetonitrile, 0.1% trifluoroacetic acid and 0.1% for-mic acid in water (v/v/v) for data acquisition. Peptides were separated using re-versed-phase liquid chromatography on a rapid separation high performance liquid chromatography (HPLC) system (Dionex, Thermo Fisher Scientific) and analysed using trapped ion mobility spectrometry with a quadrupole time-of-flight mass spectrometer (timsTOF; Bruker Daltonics, Breman, DE) coupled to a nano-electrospray ion source (CaptiveSpray, Bruker Daltonics). Samples were analysed randomly and in duplicate over a maximum time of 24 hrs at 4 °C in the autosampler. Quality controls, a combi-nation of commercial HeLa protein tryptic digests (Thermo Fisher Scientific) and in-dexed retention time peptides from an iRT Kit (Biognosys, Schlieren, CH), were run in duplicate at the beginning and again at every 10th sample in all batches. Samples and quality controls were loaded onto a 1.6 µm, 25 cm x 75 µm Aurora C18 reversed phase column (IonOpticks, Fitzroy, VIC, AU) with a 5 µL injection and 400 nL/min flow rate. A multistep liquid chromatography gradient was used with solvent A (0.1% formic acid in HPLC grade water (v/v)) and eluting solvent B (0.1% formic acid in HPLC grade acetonitrile (v/v)) over a total run time of 60 min (0 – 15 min at 2% B, 15 – 45 min at 2 – 25% B, 45 – 49 min at 25 – 95% B; 49 – 52 min at 95% B and 52 – 60 min 2% B). The nanospray ionisation, 1,800 V, was kept at 50 °C in a Sonation Column oven (Sonation GmbH, Biberach, DE). The timsTOF was operated using the default application with TIMS on and PASEF positive mode for ddaPASEF for spectral library generation and diaPASEF for plasma sample data acquisition. Radio-frequency fields for ion-funnels 1 and 2 were set to 300 and 200 Vpp, respectively. Trapped ion mobility separation was performed via a scanning mobility range of 0.6 – 1.6 1/k0. The PASEF runs were ac-quired with 100 ms ramp and 10 PASEF MS/MS scans per top10, with a cycle time of 1.32 s. The signal-to-noise ratio was increased to the summation of individual TOF scans, the target value was set to 20,000 arbitrary units and the intensity threshold was set to 2,500 arbitrary units. The mass range for MS and MS/MS spectra were set to 100 – 1,700 m/z and calibrated for mass accuracy and collision cross section prior to use. For DIA, an optimised method for long gradients were used with the mass range m/z, 400 – 1,200, divided into 32 variable windows and a scanning mobility range of 0.6 – 1.6 1/k0. The trapped ion mobility spectrometry dimension was calibrated offline using the ESI LC/MS Low Concentration Tuning Mix (Agilent Technologies, Denmark).

*Statistical analysis*

The raw diaPASEF files were processed with Spectronaut™ powered by Pulsar (version 14.10.201222.47784; Biognosys). Standard default settings were employed, including QUANT2.0 label-free quantitative using the MS2 profile. Normalised retention time-based liquid chromatography run alignment were applied using the internal peptide library hosted by Spectronaut™. All label-free quantitative data were normal-ised on the global medial and filtered by a q-value of 0.01 (equal to an FDR of 1%).

These were further processed and analyzed with the analytical software Perseus (1.6.12.0). To ensure that only values and IDs of high confidence were used for the statistical analysis, a series of filtration- and validation steps were carried out. This eliminated proteins only identified by a single peptide, and proteins present in less than 50% of all samples. To ensure more comparable results between pre- and post-treatment samples, only paired patient samples were used. All measurements were tested for normality with a histogram and screened for outliers with Pearson’s correlations (all values >0.875), boxplots, and principal component analyses (PCAs). This allowed for the use of paired students t-tests to identify significantly regulated amounts proteins (p<0.05) between the two groups.
