# Supplementary material for: Intraarticular gold for knee osteoarthritis: An ancillary analysis of biomarkers and outcome of a pilot study
Source: Osteoarthr Cartil Open. 2024 Aug 31;6(4):100514. doi: 10.1016/j.ocarto.2024.100514 (PMC11406078; doi:10.1016/j.ocarto.2024.100514)
Supplement: Multimedia component 4 [file mmc4.docx]

**Project summary**

**Rationale**

To date, it is still debated how much inflammation per se contributes to pain in osteoarthritis. A variety of studies have investigated various aspects such as synovitis but still, no clear associations to pain have been found. Many studies have attempted to modulate the effect of the inflammatory mediators on the pain receptors by e.g., interacting with the arachidonic acid pathways. As such compounds have both peripheral and central effects it is difficult to use such compounds as specific modulators of specific local inflammatory processes. So far, no compounds have been used to modulate selectively the local organelles involved in the inflammatory processes of joint pain.

**Objectives**

The purpose of this study is to investigate if specific effects can be obtained by modulating selectively macrophages and mast cells in the osteoarthritic knee joint as assessed by mechanistic pain assessment tools (quantitative sensory testing), blood and synovial fluid analysis, and clinical pain ratings in patients with painful knee osteoarthritis.

The aim of this study is to use gold particles as a model compound to modulate specifically and selectively the function of macrophages and mast cells and investigate how this modulates pain and pain sensitization, clinical outcome, and proteomic markers in synovial fluid and serum.

**Methods**

Approximately 72000, 20-40 my-meter diameter, sterilized gold particles (=20 mg) will be provided in vials (The Berlock® Gold Implants). 5-10 ml of synovial fluid is aspirated (20G needle) from the affected OA knee. The vial of gold microparticles is mixed with 2 ml synovial fluid**,** and the mix of gold and synovial fluid is injected intra-articularly into the patient’s knee. The remaining synovial fluid and blood samples are stored for future analysis.

We will include 30 patients in group I for evaluation of the modulation of macrophages and mast cells. Quantitative sensory testing, patient-reported questionnaires, clinical examination, and blood and synovial samples will be collected before and 8 weeks after treatment. At the 2-year follow-up, patient-reported questionnaires and additional treatments will be collected.

Patients who have not fulfilled the criteria for evaluation of the modulation of macrophages and mast cells will be included in group II and will be treated using gold microparticles and hyaluronic acid and followed using patient-reported questionnaires. This group will be followed for evaluation of the generalized effect on pain and function. This group will be followed before, 8 weeks after, and at 2 years using patient-reported questionnaires, and additional treatments will be collected.

**Expected outcome**

For the included 30 patients we expect a difference of 0.5 SD in the primary outcome, the patient-reported pain, and the function questionnaire WOMAC. For the total sample of included and excluded patients, we expect more than 70% to report a benefit of treatment on a global outcome measure.

**General information**

*Protocol title, protocol identifying number (if any), and date*

The effect of inflammation on pain in patients with osteoarthritis of the knee – N-20160045 – 17/7/2016

*Sponsor*

Sten Rasmussen M.D., Ph.D.

Department of orthopedic surgery, Aalborg University Hospital, Denmark

Lars Arendt-Nielsen M.Sc. Ph.D., DMSc,

Center for Neuroplasticity and Pain (CNAP), Aalborg University

*Funding*

Department of Clinical Medicine provided funding for this study. Center for Neuroplasticity and Pain (CNAP) (Lars Arendt-Nielsen) provided funding for this study. CNAP is supported by the Danish National Research Foundation (DNRF121) and the Danish Rheumatism Association (R204-A7645). The Danish National Mass Spectrometry Platform for Functional Proteomics (PRO-MS; grant no. 5072-00007B); The Obelske family foundation, the Svend Andersen Foundation, and the SparNord foundation are acknowledged for grants to the analytical platform, enabling parts of this study.

*The primary investigator and research site*

Sten Rasmussen MD PhD

Department of orthopedic surgery, Aalborg University Hospital, Denmark, 18-22 Hobrovej, DK-9000 Aalborg Denmark, Phone +45 25520462

*All investigators*

Sten Rasmussen, M.D., CBE., Ph.D., 1,2 sten@dcm.aau.dk

Kristian Kjær Petersen, M.Sc., Ph.D., 3, 4 kkp@hst.aau.dk

Christopher Aboo, M.Sc., 3 cabo@hst.aau.dk

Jakob Skallerup Andersen, M.Sc., 3 jacobsa@hst.aau.dk

Emilie Skjoldemose, stud. med., 1 e.g.skjoldemose@gmail.com

Nia Kristine Jørgensen, stud. med., 1 nia.j@rn.dk

Allan Stensballe, M.Sc., Ph.D., 3, 5 as@hst.aau.dk

Lars Arendt-Nielsen, M.Sc., Ph.D., DMSc., 3, 4, 6 lan@hst.aau.dk

1. Department of Clinical Medicine, Aalborg University
2. Department of Orthopedic Surgery, Sport and Arthroscopy, Aalborg University Hospital
3. Department of Health Science and Technology, Aalborg University.
4. Center for Neuroplasticity and Pain, Department of Health Science and Technology, Faculty of Medicine, Aalborg University, Aalborg, Denmark
5. Clinical Cancer Research Center, Aalborg University Hospital, Aalborg, Denmark
6. Department of Medical Gastroenterology, Mech-Sense, Aalborg University Hospital

*Laboratories*

Department of clinical biochemistry, Aalborg University Hospital

Department of Health Science and Technology, Aalborg University.

**Rationale & Background information**

Osteoarthritis (OA) of the knee and hip is the most common musculoskeletal joint disease worldwide. Although a major symptom of OA is chronic joint pain, which has a significant effect on patients’ quality of life, the pain mechanisms remain largely unknown.

Recently, clinical studies have suggested the existence of a neuropathic component in OA pain [5]. Accumulating evidence has been indicating that painful OA patients show peripheral and central sensitization [3,16]. Quantitative sensory testing (QST) is a relevant way to assess peripheral and central sensitization in joint pain [9]. Several of the studies have administered mechanical stimuli, and the most often used modality has been pressure. A recent review concluded that people with OA have lower Pressure Pain Thresholds (PPT), facilitated temporal summation, and impaired conditioned (CPM) compared with healthy controls [5]. Also, recent evidence has linked QST profiles to the development of chronic pain [29,38], which emphasizes the importance of studying the central nervous system.

Inflammation markers have been correlated with pain intensity [35], and systemic inflammation can lead to sensitization of peripheral nociceptors [1]. Recently it was reported that higher preoperative levels of TNF-α, MMP-13, and IL-6 in synovial fluid may indicate a smaller improvement in pain 2 years after TKR [15].

Injectable solutions of gold-thio-compounds have been used to treat rheumatoid arthritis for nearly 100 years [13,21]. Several studies suggest that gold ions reduce pain, joint swelling, and inflammation, and increase joint motility [24,31,33]. Gold salt therapy (auranofin; RidauraR) has been vetted by the FDA and approved (May 1985) and recommended by the American College of Rheumatology as a Disease Modifying Anti Rheumatic Drug - DMARD (https://www.rheumatology.org).

Gold salts, whether injected or given orally, are effective [33,37] in reducing joint pain. Studies report patients going into total remission [28] and others report a 30% reduction in symptoms [20,27]. In addition, Clark et al., 1997 [8] concluded in a Cochrane meta-analysis that systemic treatment with gold was highly effective compared to placebo.

The use of gold salt given systemically has been criticized since when gold compounds are made to circulate systemically via the circulatory system after oral or parenteral administration, the gold ions reach essentially all tissues and organs. In some patients, organs that are not involved in the arthritic disease, such as the kidneys, liver, and skin are adversely affected by gold. About 30% of patients develop such remote organ “side effects” and discontinue gold treatment [20]. In addition, newer drugs have been developed with efficacy almost equal to gold ions in many patients, and less toxic.

To overcome the toxicity, a method that captures the efficacy of the gold ions but eliminates the remote side effects have been developed[10,23]. Briefly, the method isolates the gold ions within the affected joints by injecting solid gold metal directly into the diseased joint, whereby a very slow dissolution and local diffusion deliver the therapeutic gold ions to affected areas within the joint. In this therapy, 99.99% pure gold implants are injected into the body.

Individual single beads are implanted with a 16-gauge needle based on an X-ray picture in deeply anesthetized animals close to the capsule of the affected joint(s) (videos www.goldtreat.com). Over *3*000 dogs and more than 100 horses have had this treatment for osteoarthritis at 50+ veterinary clinics and hospitals throughout Europe, (www.goldtreat.com). Success rates of 50 – 70% are quoted (www.goldtreat.com), and a double-blind, placebo-controlled study on dogs showed improvement in over 80% of the dogs, which continues for 18 months [18] as the gold particles remain in the joint[26].

The systemic concentration of released gold ions is low but clinically effective and because the gold ions do not spread in the organism, but instead stay local, the technique is safe [12]. The trials to date indicate that only one local application is needed to obtain lifelong clinical effects [12].

To date, it is still debated how much inflammation per se contributes to pain in osteoarthritis. A variety of studies have investigated various aspects such as synovitis but still, no clear associations to pain have been found[30]. Many studies have attempted to modulate the effect of the inflammatory mediators on the pain receptors by e.g., interacting with the arachidonic acid pathways[4]. As such compounds have both peripheral and central effects it is difficult to use such compounds as a specific modulator of specific local inflammatory processes. So far, no compounds have been used to modulate selectively the local organelles involved in the inflammatory processes of joint pain.

Gold particles seem as the only known method to obtain such a local effect on one specific element involved in the inflammatory process – the macrophages and the mast cells.

If a foreign object gets embedded in the body, macrophages will attack the object and digest it. If the foreign object has a diameter bigger than 20 microns, the macrophage cannot engulf it. Instead, the macrophages create a membrane on the surface of the foreign body and start a chemical attack in order to dissolve it. If the foreign body consists of gold the macrophages will cause the release of gold ions from the foreign body.

Gold ions, taken up by the macrophages, cause them to malfunction[10,11,23]. As the macrophage is a ‘conductor’ of the inflammatory process it causes a drastic restraint of the inflammation and finally brings it to a stop[22,25]. The released gold ions will be taken up by the macrophages themselves, but also diffuse out into the surrounding tissue where they are taken up by other cells as well e.g. mast cells and other connective tissue cells[7,32]. When the gold ions accumulate in the secretory granules of mast cells it blocks histamine release and thereby decreasing local edema, and additionally suppressing pain.

The amount of bio-released gold ions is related to the intensity of inflammation. Only a few gold ions are released in immunological nonreactive tissue. This means that if a local inflammation that has been treated with gold implants, the increase in macrophages will cause an immediate inhibition of the inflammation.

The combined effect of gold ions on macrophages and mast cells is together believed to cause a significant reduction in pain following treatment with metallic gold particles. If this principle can be validated, it may provide important information for the development of new and better treatments for e.g., joint pain.

Recently, Seifert et al.[34] called for studies providing new insights into the mode of action of gold ions and suggest investigating the effects on key mechanisms involved in arthritis or other inflammatory conditions.

The aim of this study is to use gold particles as a model compound to modulate specifically and selectively the function of macrophages and mast cells and investigate how this modulates pain and pain sensitization in the osteoarthritic knee assessed by mechanistic pain assessment technologies (QST).

**Study goals and objectives**

The purpose of this study is to investigate if specific effects can be obtained by modulating selectively macrophages and mast cells in the osteoarthritic knee joint as assessed by mechanistic pain assessment tools (quantitative sensory testing), blood and synovial fluid analysis, and clinical pain ratings in patients with painful knee osteoarthritis.

Hypothesis: Modulating macrophages and mast cells by gold microparticles injected into the osteoarthritic knee joint will modulate inflammation specifically, inhibit pain sensitisation and provide pain relief to patients with knee OA.

The aim of this study is to use gold particles as a model compound to modulate specifically and selectively the function of macrophages and mast cells and investigate how this modulates pain and pain sensitization, clinical outcome, and proteomic markers in synovial fluid and serum.

**Study design**

**Patients**

***Group 1***

For this part of the study 30 patients with single knee OA will be included using a narrow set of inclusion and exclusion criteria for evaluation of the modulation of macrophages and mast cells.

The following inclusion and exclusion criteria will be used.

Patients with radiographically confirmed KOA (Kellgren-Lawrence grade ≥ 2), pain for more than three months, and maximal pain intensity VAS (Visual Analogue Scale, 0-10) ≥ 5 during the last week, and with knee joint effusion on MRI that can be aspirated. Eligible patients were enrolled at the specialized, public outpatient clinic at Aalborg University Hospital, Denmark. The exclusion criteria were: 1) active adjuvant treatment for any malignancy, 2) active infection and antibiotic treatment, 3) active treatment with steroids, biological or other anti-rheumatic medication, 4) chronic pain and other than knee joint pain, 5) inability to comply with the protocol and, 6) inadequacy in written and spoken national language.

***Group 2***

During the inclusion period for group 1, we will include a group of patients with minor degree of knee OA, chronic pain, and other pain than knee joint pain (multisite pain) for evaluation of the generalized effect on pain and function.

The following inclusion and exclusion criteria will be used.

Patients with radiographically confirmed KOA (Kellgren-Lawrence grade ≥ 1), pain for more than three months, and maximal pain intensity VAS (Visual Analogue Scale, 0-10) ≥ 5 during the last week. Eligible patients were enrolled at the specialized, public outpatient clinic at Aalborg University Hospital, Denmark. The exclusion criteria were 1) active adjuvant treatment for any malignancy, 2) active infection and antibiotic treatment, 3) active treatment with steroids or biological medication, 4) inability to comply with the protocol and 5) inadequacy in written and spoken national language.

The expected duration of the study is 4-5 years with an inclusion period of two years and a follow-up time of two years.

**Methodology**

Approximately 72000, 20-40 my-meter diameter, sterilized gold particles (20 mg, sterile 99,99% pure gold microparticles) will be provided in vials (The Berlock® Gold Implants) for both groups.

***Group 1***

5-10 ml of synovial fluid is aspirated (20G needle) from the affected OA knee. The vial of gold microparticles is mixed with 2 ml synovial fluid**,** and the mix of gold and synovial fluid is injected intra-articularly into the patient’s knee. The remaining synovial fluid and additional blood samples are collected and stored for future analysis.

***Group 2***

The vial of gold microparticles is mixed with 2 ml hyaluronic acid (Suplasyn®, 20mg/2ml**,** and the mix of gold and hyaluronic acid is injected intra-articularly into the patient’s knee.

Patients in group I for evaluation of the modulation of macrophages and mast cells. Quantitative sensory testing, patient-reported questionnaires, clinical examination, and blood and synovial samples will be collected before and 8 weeks after treatment. At the 2-year follow-up, patient-reported questionnaires and additional treatments will be collected.

Patients in group II for evaluation of the generalized effect on pain and function will be treated using gold microparticles and hyaluronic acid will be followed using patient-reported questionnaires. This group will be followed before, 8 weeks after, and at 2 years using patient-reported questionnaires, and additional treatments will be collected.

***Clinical pain and function assessment***

WOMAC Pain, function and stiffness score

The Western Ontario and McMaster Universities Arthritis Index WOMAC [6] is a subject-rated instrument that measures Osteoarthritis (OA) symptoms. The questionnaire contains 5 pain questions, 2 stiffness questions, and 17 physical function questions (24 questions total). Each question utilizes a 5-point scale, from 0 (none) to 4 (extreme). The WOMAC score will be used before treatmen and 8 weeks and 2 years after.

Weekly Pain Diary

Subjects rated their pain intensity on a VAS scale every day at home. On the VAS scale ‘‘0” indicates ‘‘no pain”, and ‘‘10” indicates ‘‘maximal pain”. Subjects rated the pain severity for Average Pain Score (APS) for the last 24 hours, worst pain for the last 24 hours, and pain severity at night. The weekly pain diary will be used for 8 weeks after treatment.

Global Rating of Change Scale

Using the Global Rating of Change Scale, we asked the question, “Concerning your knee, how will you describe yourself compared to immediately before the injection of gold into your knee” and evaluated the answer on an 11-point scale from very much worse (-5) to complete recovered (5) with a score of “0” indicating no changes. The global rating scale will be used 8 weeks and 2 years after treatment.

Pain Detect questionnaire (PDQ)

The PDQ is a validated, easy to use screening tool that predicts the likelihood of a neuropathic pain component in chronic pain disorders[14]. It shows higher sensitivity and specificity in comparison with other neuropathic pain screening questionnaires. The questionnaire is comprised of 3 major components: gradation of pain, pain course pattern and radiating pain. There are 7 questions evaluating gradation of pain. Each question is scored by the patient using a 0 to 5 score with 0 = never, 1 = hardly notice, 2 = slightly, 3 = moderately, 4 = strongly and 5 = very strongly. There is one question evaluating pain course patterns. Patients select from one of four pictures to indicate which pattern of pain best describes their course of pain. Each picture is associated with a unique score of 0, -1, or +1 (2 pictures have this score possible). There is one question evaluating radiating pain with a yes (score of +2) or no (score of 0) response option. The PD-Q is calculated by the addition of the patient’s responses to all questions. Thus, the maximum possible score is 38, the minimum possible score is -1. Only integral values are possible. Total PD‑Q scores of ≤ 12 indicate that a neuropathic pain component is unlikely (< 15%). Total PD‑Q scores of ≥ 19 indicate that a neuropathic pain component is likely (>90%). Scores of 13 to 18 are uncertain.

***Quantitative Sensory Testing (QST)***

QST will be performed before and 8 weeks after treatment

*Pressure Pain Sensitivity*

A handheld algometer (Somedic, Hörby, Sweden) with a 1-cm^2^ probe (covered by a disposable latex sheath) is used to record the pressure pain threshold (PPT) on 7 sites at the affected knee with two distant sites at the tibialis anterior muscle (TA, 5 cm distal to the tibial tuberosity) and at the extensor carpi radialis longus muscle (arm, 5 cm distal to the lateral epicondyle of humerus). The sites in the peripatellar regions will be: 2 cm distal to the inferior medial edge of patella (Site 1); 2 cm distal to the inferior lateral edge of patella (Site 2): 3 cm lateral to the midpoint of the lateral edge of patella (Site 3); 2 cm proximal to the superior lateral edge of patella (Site 4): 2 cm proximal to the superior edge of patella (Site 5); 2 cm proximal to the superior medial edge of patella (Site 6); 3 cm medial to the midpoint of the medial edge of patella (Site 7). An interval of minimum 20 seconds is kept between each PPT assessment. The PPT is defined to the subject as ‘‘the point at which the pressure sensation just becomes painful.’’ Pressure is increased gradually at a rate of 30 kPa/s until the pain threshold is reached and the subject presses a button.

*Cuff Pressure Algometry*

Deep-tissue pain sensitivity will be evaluated by cuff pressure stimuli using a computer-controlled cuff algometer (NociTech and Aalborg University, Denmark) including a 13-cm wide tourniquet cuff (VBM, Sulz, Germany) and an electronic VAS (Aalborg University, Denmark) for recording of the pain intensity. The cuff will be placed at the level of the head of the gastrocnemius muscle of the leg most affected by knee OA. The electronic continuous VAS (sliding resistor) is 10 cm long and sampled at 10 Hz; 0 cm indicated “no pain” and 10 cm indicated “maximum pain”.

The cuff is automatically inflated (inflation rate: 1 kPa/s). The subject is instructed to rate the pain intensity continuously on the VAS from the first sensation of pain (pressure detection threshold, PDT) and to press the release button at “the pain intensity strong enough to make one feel like interrupting or stopping it”. The pressure value at the termination of pressure inflation is defined as the pressure-pain tolerance (PTT) and the corresponding VAS-score is defined as the pressure-pain limit.

A total of 10 repeated mechanical pressure stimuli will be delivered at 0.5 Hz (1 s stimulus duration and 1 s interval between stimuli) to the lower leg to evaluate temporal summation. A constant pressure between the individual pressure stimuli of 1 kPa will be applied to avoid movement of the cuff. During the 10 repeated stimuli, the patients will be instructed to continuously rate the pain intensity on a 10 cm continuous VAS (sliding resistor) (“0” represented “no pain”, and “10” represented “maximal pain”).

A painful conditioned stimulus will be applied to the contralateral lower leg simultaneously; assessment of PDT and PTT will be performed using a single chamber cuff on the ipsilateral lower leg (test stimulus) as a measure of CPM. The conditioned stimulus will be terminated right after the PDT and PTT are assessed.

**Sample collection and biobanking**

SF from the knees will be centrifugated for 10 minutes at 2200G to remove cell debris, and the supernatant frozen in aliquots at -80℃ for future analysis. Besides, a 6 ml venous whole blood sample was taken and centrifugated at 3000 RPM for 15 minutes. The serum fraction will be stored at -80℃ for future analysis. All sample data will be collected and kept following Danish legislation on data handling at the Department of Biomedicine at Aalborg University Hospital. Each patient sample will be divided into aliquots to avoid unnecessary freezing and thawing cycles.

Cell free DNA will be determined in SF and serum by Quant-iT™ PicoGreen™ dsDNA Assay Kit (ThermoFisher Scientific) according to the manufacture’s instruction (Birkelund et al., 2020). A Proximity Extension Assay (PEA) will be carried out essentially according to Giordano et 2020 on representative SF samples (n=6) to quantify low abundance affected by the gold treatment essentially. Sample preparation for proteome analysis will be prepared essentially as Birkelund (Birkelund et al., 2020). The protein concentration in biofluids will be measured by a microassay. A total of 25ug SF and serum subject to in-situ trypsinization (trypsin ratio 1:100) using reduction and alkylation using a Filter Aided Sample Preparation protocol (10kDa cut-off) before analysis with tandem mass spectrometry (MS/MS). To expand the protein coverage of the MS analysis, a pH-fractioning of the enzymatically digested samples will be carried out. A total of 500ng tryptic digest will be analyzed in technical duplicates in randomized order using a nano UPLC ESI MS/MS setup consisting of a Dionex RSLC nano pump (Dionex RSLC, Thermo Fisher Scientific) connected to a Bruker timsTOF PRO mass spectrometer operated in DIA-PASEF positive ion mode (Bruker Daltronics, DE).

*Proteomic measures*

The analyses of the proteomic primary raw data output will be searched against spectral databases generated by fractionated pooled samples using human reference proteome database (05.2021; 20364 entries) in Biognosys Spectronaut (v. 14.10) and using statistical analysis. Quantitative proteome analysis of SF and serum samples will be accomplished by discovery proteomics using DIA-PASEF. Functional association of significantly regulated proteins in SF and serum with gene onthology and function will be assessed by Metascape

**Procedure**

After signing the informed consent patients will be investigated clinically and included/excluded according to the criteria. If included, they are invited back to the clinic. The clinical and experimental measures will be assessed at baseline. The patients will receive the intraarticular gold injection in the affected study knee. Patients will come back to the clinic after 8 weeks where all the measures will be taken. During the 8 weeks period the patients will fill in the daily pain diary and medication used. Patients will come back for a 2-year follow measuring clinical pain and function.

**Risks, side effects and disadvantages**

The pain assessment methods used are well-proven and all are performed in accordance with standard procedures previously used in similar studies. There are no reports from our or other institutions on long-term side effects of the methods. The applied stimulation can be interrupted at any time. The subjects will be under observation during the trial.

Patients will experience weak and short-term pain during quantitative sensory testing (QST). Specifically, the pressure stimulation will cause a pressing or clamping pain however, the pain disappears immediately after the end of the influences and does not cause any side effects.

Intraarticular injection of the small volume of gold particles are not expected to give any residual side effects as intraarticular injection is a standard clinical procedure. Patients may experience some pain associated with the injection, which is a normal reaction. The injection site is properly sterilized according to the clinical standard to minimize the possibility of inducing an infection.

Injection of gold is used in different settings for treatment of articular pain.

**Study shortcomings**

The study is not investigating the effect of the gold injection as such but the possible role of modulating specific inflammatory mechanisms, pain, and function. Therefore, the study is not including a placebo control.

**Statistical analysis**

This present exploratory study will investigate whether gold ions have a clinical role in treating KOA. Based on preclinical evidence, we estimated medium effect size (Cohen’s d = 0.5), and with a power of 80% and a significant level at 0.05, a sample size calculation estimated a total sample of 27 subjects will be needed in group 1. A total of 30 subjects will be included to account for possible drop-out.

The clinical outcome measures analysis will be performed using the Stata software, version 16.0 (StataCorp.) ANOVA will be used for the analysis of repeated measures of pain, the pain diary, and QST parameters. Wilcoxon test will be used for all other measurements.

**Ethical considerations**

The study complies with the Helsinki Declaration and is submitted to the North Denmark Region Committee on Health Research Ethics for approval. The methods used have been tested and performed in several studies both in Denmark and abroad and no long-term side effects have been reported.

Patients with (OA) may eventually suffer from chronic pain leading to disabilities of the individual and associated costs for public health care systems [19]. OA is the most frequent painful musculoskeletal condition in the elderly population [26]. Recent evidence suggests that QST profiles are associated with the development of chronic pain [29,38], why studying QST profiles in patients with OA can be crucial for new pain relieving treatments.

The current study will introduce mild and short-lasting pain to the study participants but the outcome from this study can lead to a better understanding of the central nervous system, which can lead to better treatments.

The study will follow the principles of the Declaration of Helsinki and was approved by the local ethics committee of the North Denmark Region by 27/07/2016 (N-20160045). The regional data protection agency approved the project by 06/07/2016 (2008-58-0028, ID 2016-116) and registered in ClinicalTrial.Gov (NCT03389906).

Approval for this study was waived be the Danish Food and Drug Administration since gold is a primary element, not a medication, and not an implant by 10/04/2016 (LMST2016033454).

**Insurance**

The subjects are covered by the Danish Patient Insurance Association (Patienterstatningen).

**Personal data**

Data will be stored after termination of the project. These data can only be used for the interpretation of this project and will therefore not be of interest to third party. Data are stored in accordance with the stipulations in The Danish Personal Data Protection Act (Persondataloven) and other relevant Danish legislation. The project is reported to The Danish Data Protection Agency by 06/07/2016 (2008-58-0028, ID 2016-116); “Bekendtgørelse om ændring af bekendtgørelse om undtagelse fra pligten til anmeldelse af visse behandlinger, som foretages for en privat dataansvarlig”.

**Project economy**

These studies are initiated by Professor Sten Rasmussen, Department of Clinical Medicine, Aalborg University, and Professor Lars Arendt-Nielsen, Center for Neuroplasticity and Pain, Department of Health Science and technology, Aalborg University.

Department of Clinical Medicine provided funding for this study. Center for Neuroplasticity and Pain (CNAP) (Lars Arendt-Nielsen) provided funding for this study. CNAP is supported by the Danish National Research Foundation (DNRF121) and the Danish Rheumatism Association (R204-A7645). The Danish National Mass Spectrometry Platform for Functional Proteomics (PRO-MS; grant no. 5072-00007B); The Obelske family foundation, the Svend Andersen Foundation, and the SparNord foundation are acknowledged for grants to the analytical platform, enabling parts of this study.

**Payment to subjects**

Patients receive no payment for their participation in the study.

**Publishing of results**

The results of the project will be published in recognised journals regardless of the outcome of the project.

**Time schedule**

The experimental study will start after approval and be completed in January 2021

## Guidelines for Oral information and Informed Consent

### *Summoning Potential Subjects*

When contacting potential subjects in the outpatient clinic the following should be stated:

- That it is a request for participation in a scientific research project
- The purpose of the project
- That participation is voluntary and that the subject can withdraw from the project at any time without consequences
- That the potential subject has time to consider his/her participation before giving consent to participation in the project and that the potential subject is welcome to bring a family member or a friend to the information meeting. The potential volunteer will receive the leaflet “The Rights of a Trial Subject in a Health Scientific Research Project”/ "Forsøgspersonens rettigheder i et sundhedsvidenskabeligt forskningsprojekt" which includes information on confidentiality, right of access to documents and right to complain.
- That the material “Information for Participants”/”Deltagerinformation” will be forwarded by mail/e-mail to the potential subject in order for him/her to know more about the project before the information meeting.
- Finally, time for the information meeting is arranged

### ***The Information Meeting***

The information meeting is held in a quiet room where it is possible to have an uninterrupted conversation. Coffee/tea/soft drink may be served. The information meeting is held by a senior researcher who has been authorized to do the information.

The meeting is to include the following information/questions:

- Participation is voluntary and the subject can withdraw from the project at any time without consequences
- The subject has time to consider his/her participation before giving consent to participation in the project, and the subject is welcome to bring a family member or a friend to the information meeting.
- The subject is asked whether he/she wants a family member/friend to be present at the meeting.
- The purpose of the experiment is presented, and it is explained how the experiment is performed. The “Information for Participants”/”Deltagerinformation”, which has been sent to the potential subject in advance, is the starting point for the information meeting.
- The subject is asked if he/she is healthy or whether he/she has an infectious disease.
- The subject is asked whether he/she is a Danish citizen. If the answer is no, he/she is asked if he/she has a valid work permit.
- The leaflet “The Rights of a Trial Subject in a Health Scientific Research Project”/ "Forsøgspersonens rettigheder i et sundhedsvidenskabeligt forskningsprojekt" is handed over. It is explained that it includes information on confidentiality, right of access to documents and right to complain.
- The subject is asked whether he/she has read “Information for Participants”/ ”Deltagerinformation”. If this is not the case, we will ask the subject to read it.
- When it has been ensured that the subject has read the “Information for Participants”/”Deltagerinformation”, he/she is asked whether he/she has questions about the experiment.
- After this a demonstration is given in the lab; measuring equipment and its use is presented to the subject.
- It is underlined that participation is voluntary, and that the subject has time to consider his/her participation (please note that The National Committee on Health Research Ethics recommends 24 hours of deliberation time)
- Again, it is underlined that participation is voluntary and that the subject can withdraw his/her consent at any time without consequences.
- The subject is informed that if he/she does not need time to consider the participation, the consent can be given at the information meeting.
- Time/place for the experiment is agreed.
- Finally, information about the contact person of the experiment is given (it is shown to the subject that the name and contact details appear from the “Information for Participants”/”Deltagerinformation”) and it is informed that this person can be contacted at any time if further questions should arise.

**References**

[1] Andrew D, Greenspan JD. Mechanical and heat sensitization of cutaneous nociceptors after peripheral inflammation in the rat. J Neurophysiol 1999;82:2649-2656.

[2] Arendt‐Nielsen L, Egsgaard L, Petersen K, Eskehave T, Graven‐Nielsen T, Hoeck H, Simonsen O. A mechanism‐based pain sensitivity index to characterize knee osteoarthritis patients with different disease stages and pain levels. European Journal of Pain 2015.

[3] Arendt-Nielsen L, Nie H, Laursen MB, Laursen BS, Madeleine P, Simonsen OH, Graven-Nielsen T. Sensitization in patients with painful knee osteoarthritis. Pain 2010;149:573-581.

[4] Arendt-Nielsen L, Egsgaard LL, Petersen KK. Evidence for a central mode of action for etoricoxib (COX-2 Inhibitor) in patients with painful knee osteoarthritis. Pain 2016.

[5] Arendt-Nielsen L, Skou ST, Nielsen TA, Petersen KK. Altered Central Sensitization and Pain Modulation in the CNS in Chronic Joint Pain. Current Osteoporosis Reports 2015:1-10.

[6] Bellamy N, Buchanan WW, Goldsmith CH, Campbell J, Stitt LW. Validation study of WOMAC: a health status instrument for measuring clinically important patient relevant outcomes to antirheumatic drug therapy in patients with osteoarthritis of the hip or knee. J Rheumatol 1988;15:1833-1840.

[7] Christensen MM, Danscher G, Ellermann-Eriksen S, Schiønning JD, Rungby J. Autometallographic silver-enhancement of colloidal gold particles used to label phagocytic cells. Histochemistry 1992;97:207-211.

[8] Clark P, Tugwell P, Bennett Kathryn J, Bombardier C, Shea B, Wells George A. Injectable gold for rheumatoid arthritis. Cochrane Database of systematic reviews 1997.

[9] Courtney CA, KAVCHAK AE, Lowry CD, O'HEARN MA. Interpreting Joint Pain: Quantitative Sensory Testing in Musculoskeletal Management. J Orthop Sports Phys Ther 2010;40:818-825.

[10] Danscher G. In vivo liberation of gold ions from gold implants. Autometallographic tracing of gold in cells adjacent to metallic gold. Histochem Cell Biol 2002;117:447-452.

[11] Danscher G, Larsen A. Effects of dissolucytotic gold ions on recovering brain lesions. Histochem Cell Biol 2010;133:367-373.

[12] Danscher G, Stoltenberg M. Silver enhancement of quantum dots resulting from (1) metabolism of toxic metals in animals and humans,(2) in vivo, in vitro and immersion created zinc–sulphur/zinc–selenium nanocrystals,(3) metal ions liberated from metal implants and particles. Prog Histochem Cytochem 2006;41:57-139.

[13] Forestier J. Rheumatoid arthritis and its treatment by gold salts. The lancet 1934;224:646-648.

[14] Freynhagen R, Baron R, Gockel U, Tölle TR. Pain DETECT: a new screening questionnaire to identify neuropathic components in patients with back pain. Current Medical Research and Opinion® 2006;22:1911-1920.

[15] Gandhi R, Santone D, Takahashi M, Dessouki O, Mahomed NN. Inflammatory predictors of ongoing pain 2 years following knee replacement surgery. The Knee 2013;20:316-318.

[16] Graven‐Nielsen T, Wodehouse T, Langford R, Arendt‐Nielsen L, Kidd B. Normalisation of widespread hyperesthesia and facilitated spatial summation of deep‐tissue pain in knee osteoarthritis patients after knee replacement. Arthritis & Rheumatism 2012;64:2907-2916.

[17] Guy W, Bonato RR. Manual for the ECDEU assessment battery. : US Department of Health, Education, and Welfare, National Institute of Mental Health, 1970.

[18] Jæger GT, Larsen S, Søli N, Moe L. Two years follow-up study of the pain-relieving effect of gold bead implantation in dogs with hip-joint arthritis. Acta Vet Scand 2007;49:1.

[19] Jinks C, Jordan K, Croft P. Osteoarthritis as a public health problem: the impact of developing knee pain on physical function in adults living in the community: (KNEST 3). Rheumatology (Oxford) 2007;46:877-881.

[20] Jones G, Brooks PM. Injectable gold compounds: an overview. Br J Rheumatol 1996;35:1154-1158.

[21] Kean TA. Rheumatoid Arthritis and Gold Salts Therapy. Ulster Med J 1934;3:284-289.

[22] Labens R, Lascelles BDX, Charlton AN, Ferrero NR, Van Wettere AJ, Xia X, Blikslager AT. Ex vivo effect of gold nanoparticles on porcine synovial membrane. Tissue barriers 2013;1:e24314.

[23] Larsen A, Kolind K, Pedersen DS, Doering P, Pedersen MØ, Danscher G, Penkowa M, Stoltenberg M. Gold ions bio-released from metallic gold particles reduce inflammation and apoptosis and increase the regenerative responses in focal brain injury. Histochem Cell Biol 2008;130:681-692.

[24] Lehman AJ, Esdaile JM, Klinkhoff AV, Grant E, Fitzgerald A, Canvin J. A 48‐week, randomized, double‐blind, double‐observer, placebo‐controlled multicenter trial of combination methotrexate and intramuscular gold therapy in rheumatoid arthritis: Results of the METGO study. Arthritis & Rheumatism 2005;52:1360-1370.

[25] Leonavičienė L, Kirdaitė G, Bradūnaitė R, Vaitkienė D, Vasiliauskas A, Zabulytė D, Ramanavičienė A, Ramanavičius A, Ašmenavičius T, Mackiewicz Z. Effect of gold nanoparticles in the treatment of established collagen arthritis in rats. Medicina (Kaunas) 2012;48:91-101.

[26] Lie KI, Jaeger G, Nordstoga K, Moe L. Inflammatory response to therapeutic gold bead implantation in canine hip joint osteoarthritis. Vet Pathol 2011;48:1118-1124.

[27] Meier FM, Frerix M, Hermann W, Müller-Ladner U. Current immunotherapy in rheumatoid arthritis. Immunotherapy 2013;5:955-974.

[28] Menninger H, Herborn G, Sander O, Blechschmidt J, Rau R. A 36 month comparative trial of methotrexate and gold sodium thiomalate in the treatment of early active and erosive rheumatoid arthritis. Br J Rheumatol 1998;37:1060-1068.

[29] Petersen KK, Arendt-Nielsen L, Simonsen O, Wilder-Smith O, Laursen MB. Presurgical assessment of temporal summation of pain predicts the development of chronic postoperative pain 12 months after total knee replacement. Pain 2015;156:55-61.

[30] Petersen KK, Siebuhr AS, Graven-Nielsen T, Simonsen O, Boesen M, Gudbergsen H, Karsdal M, Bay-Jensen AC, Arendt-Nielsen L. Sensitization and Serological Biomarkers in Knee Osteoarthritis Patients With Different Degrees of Synovitis. Clin J Pain 2015.

[31] Rau R, Herborn G, Menninger H, Sangha O. Radiographic outcome after three years of patients with early erosive rheumatoid arthritis treated with intramuscular methotrexate or parenteral gold. Extension of a one-year double-blind study in 174 patients. Rheumatology (Oxford) 2002;41:196-204.

[32] Sadauskas E, Jacobsen NR, Danscher G, Stoltenberg M, Vogel U, Larsen A, Kreyling W, Wallin H. Biodistribution of gold nanoparticles in mouse lung following intratracheal instillation. Chem Cent J 2009;3:16.

[33] Sanders M. A review of controlled clinical trials examining the effects of antimalarial compounds and gold compounds on radiographic progression in rheumatoid arthritis. J Rheumatol 2000;27:523-529.

[34] Seifert O, Matussek A, Sjögren F, Geffers R, Anderson CD. Gene expression profiling of macrophages: implications for an immunosuppressive effect of dissolucytotic gold ions. J Inflamm 2012;9:1.

[35] Stürmer T, Brenner H, Koenig W, Günther K. Severity and extent of osteoarthritis and low grade systemic inflammation as assessed by high sensitivity C reactive protein. Ann Rheum Dis 2004;63:200-205.

[36] Turk DC, Dworkin RH, Allen RR, Bellamy N, Brandenburg N, Carr DB, Cleeland C, Dionne R, Farrar JT, Galer BS. Core outcome domains for chronic pain clinical trials: IMMPACT recommendations. Pain 2003;106:337-345.

[37] Williams HJ. Comparisons of sulfasalazine to gold and placebo in the treatment of rheumatoid arthritis. J Rheumatol Suppl 1988;16:9-13.

[38] Wylde V, Sayers A, Lenguerrand E, Gooberman-Hill R, Pyke M, Beswick AD, Dieppe P, Blom AW. Preoperative widespread pain sensitization and chronic pain after hip and knee replacement: a cohort analysis. Pain 2015;156:47.

 [39]Clausen BH, Degn M, Sivasaravanaparan M, et al. Conditional ablation of myeloid TNF increases lesion volume after experimental stroke in mice, possibly via altered ERK1/2 signaling. Sci. Rep. 2016;6:29291
